# Evaluation of ultra-low input RNA sequencing for the study of human T cell transcriptome

**DOI:** 10.1038/s41598-019-44902-z

**Published:** 2019-06-11

**Authors:** Jingya Wang, Sadiye Amcaoglu Rieder, Jincheng Wu, Susana Hayes, Rebecca A. Halpin, Melissa de los Reyes, Yashaswi Shrestha, Roland Kolbeck, Rajiv Raja

**Affiliations:** 1 Translational Science and Experimental Medicine, Early Respiratory, Inflammation and Autoimmunity, R&D Biopharmaceuticals, AstraZeneca, Gaithersburg USA; 2 Biosciences, Early Respiratory, Inflammation and Autoimmunity, R&D Biopharmaceuticals, AstraZeneca, Gaithersburg USA; 3 Research Bioinformatics, MedImmune AstraZeneca, Gaithersburg, USA; 4 Translational Medicine, Oncology R&D, AstraZeneca, Gaithersburg, USA; 5 Respiratory, Inflammation and Autoimmunity, MedImmune AstraZeneca, Gaithersburg, USA; 6 Present Address: Novartis Institutes for BioMedical Research, Cambridge, USA

**Keywords:** T cells, Next-generation sequencing

## Abstract

Deeper understanding of T cell biology is crucial for the development of new therapeutics. Human naïve T cells have low RNA content and their numbers can be limiting; therefore we set out to determine the parameters for robust ultra-low input RNA sequencing. We performed transcriptome profiling at different cell inputs and compared three protocols: **S**witching **M**echanism **a**t 5′ End of **R**NA **T**emplate technology (SMART) with two different library preparation methods (Nextera and Clontech), and AmpliSeq technology. As the cell input decreased the number of detected coding genes decreased with SMART, while stayed constant with AmpliSeq. However, SMART enables detection of non-coding genes, which is not feasible for AmpliSeq. The detection is dependent on gene abundance, but not transcript length. The consistency between technical replicates and cell inputs was comparable across methods above 1 K but highly variable at 100 cell input. Sensitivity of detection for differentially expressed genes decreased dramatically with decreased cell inputs in all protocols, support that additional approaches, such as pathway enrichment, are important for data interpretation at ultra-low input. Finally, T cell activation signature was detected at 1 K cell input and above in all protocols, with AmpliSeq showing better detection at 100 cells.

## Introduction

T cells are key players within the adaptive immune system, and their roles in health and disease have been extensively studied^1^. There are different types of T cells (such as helper, effector, cytotoxic, memory, regulatory, and gamma delta), and understanding their biology is crucial in developing new therapeutics^2^. Recently, T cells have been a target of several successful immuno-oncology drugs and enhancement of their biological activity in the context of cancer is essential in destroying tumor cells^3^. In autoimmune diseases, T cells are over-reactive and they attack the body’s own tissues and cells^4^. Therapeutic antibodies and small molecules have been developed with some success to block the deleterious actions of T cells in autoimmunity^5^. While there have been many advances in understanding T cell biology and modulating their responses in different disease settings, further studies are needed for better targeted therapeutics.

Understanding the transcriptome profiles of human T cells is important in deciphering their biology, and recent advances in RNA sequencing technology have been significant towards this goal. T cells are challenging for transcriptome profiling since their average RNA content is ~1–2 pg/cell, which can be approximately 10 fold less than rapidly proliferating cells, such as cancer cells (based on internal unpublished data). In some cases, such as studying tumor infiltrating primary lymphocytes, the number of primary T cells recovered is as low as 100, and standard RNA sequencing experiments that require at least ~200 ng of RNA (i.e. equivalent to 10^5^–10^6^ T cells) could be challenging. In this paper, we evaluated the input gradient of naïve CD4+ T cells from 100, 1000 (1 K), 5000 (5 K) to 100,000 cells and provided a comprehensive evaluation of two main transcriptome sequencing technologies appropriate for ultra-low input RNA. In addition as proof-of-concept, we validated an *in vitro* T cell activation signature using differential gene expression based on these protocols.

Multiple protocols have been developed for transcriptome profiling from very low amount of RNA inputs. Studies have been published evaluating the performance of these protocols such as Ovation (Nugen), SMARTer (Clonetech), DP-seq and CEL-seq which provided valuable insights on advantages and disadvantages of each protocol and practical considerations when performing ultra-low input RNA sequencing^6–11^. These protocols are based on unbiased sequencing of the whole cDNA pools that sequence and map all cDNA fragments to the reference transcriptome, and expression is measured by counting the total number of fragments mapping to each transcript. As technologies advanced, new protocols were developed such as AmpliSeq (Thermo Fisher) that utilizes a targeted transcriptome approach. AmpliSeq utilizes PCR assays specific for each gene being targeted, and a short amplicon is amplified and quantified to measure gene expression. This platform has shown satisfactory performance in standard RNA sequencing experiments^12^. However, no direct comparison have been made between whole transcriptome vs. targeted transcriptome profiling using ultra-low RNA inputs.

Towards this goal, we compared three different protocols based on two distinct technologies that were suitable to profile whole transcriptome from low input RNA. We used SMART-Seq v4 from Clontech which incorporates the SMART technology. SMART technology enriches for full length cDNA and subsequently improves 5′ representation. Infact, even an older version of the SMART-Seq (version 2) protocol showed the highest 5′ and 3′ coverage, and lowest ribosomal RNA content when low quality and low quantity RNA input technologies were compared^6^. In addition, the SMART technology has also been implemented for single-cell RNA sequencing due to its lower input limit of 10 pg^6^. The SMART-Seq v4 provides two Illumina-compatible options for library preparation, which mainly differ with respect to time taken as well as cDNA fragmentation method. Clontech’s low input library prep protocol involves mechanical shearing of cDNA for 200–500 bp using Covaris, while NexteraXT is a shorter method compared to Clontech and requires enzymatic digestion resulting in a slightly longer fragment sizes (~600 bp). As a comparison to whole transcriptome approach of Clontech’s SMART-Seq technology, we used targeted transcriptome approach of Thermo Fisher’s Ion AmpliSeq technology. AmpliSeq is more commonly used for targeted panels of various complexities to amplify genomic DNA^13^. We reasoned that a targeted representation of the transcriptome may enable us to maintain diversity, which is key for low input profiling methods.

In this study, we observed that as the cell input decreased the number of detected genes (DGs) decreased in SMART technology with both library preparation protocols. On the other hand, the number of DGs was comparable for all cell inputs with AmpliSeq technology. Overall, the number of DGs was not dependent on the transcript length, and the highest impact was seen on the loss of low expressing genes. Comparing technical replicates and cell inputs, there was consistent reproducibility at 1000 cell input and above with greater variability at 100 cell input. However, at 100 cell input, AmpliSeq still had higher reproducibility between technical replicates and different cell inputs than SMART technology. Deeper look at differentially expressed genes (DEGs) showed that there was decent overlap between different protocols in detecting consistent fold change. The majority of platform specific genes had high variance but was confirmed with qRT-PCR. At the lowest input of 100 cells, all protocols retained high precision; however, there was a significant drop in sensitivity in detecting DEGs. Overall, the sensitivity for DEG detection was better with AmpliSeq technology, especially at 5 K input and below. For instances in which low cell numbers are used as input, we recommend that further interrogation such as pathway analysis is performed in order to interpret the data accurately. Finally, well established T cell activation signature was detected at 1 K cell input and above with both protocols; with AmpliSeq detecting significantly higher number of these genes at 100 cell input.

## Results

### Number of detected genes decreased with reduced input in SMART technology, while it remained constant for AmpliSeq technology

First we tested three different RNA extraction kits for different cell inputs of primary human naïve CD4 T cells purified from fresh peripheral blood of healthy human volunteers. We compared PicoPure, Zymogen and Qiagen RNeasy micro kit with cell inputs of 5 K, 1 K and 100 cells from three donors (Fig. S1a). We performed qRT-PCR for two house-keeping genes: *GAPDH* and *ACTB* (β-actin) and demonstrated that Qiagen RNeasy micro kit and PicoPure kits provided the lowest CT values with highest consistency across three donors, especially at lowest input of 100 cells. At 100 cell input and 1 K cell input, PicoPure kit had more variability for one of the three donors tested, and CT values for Zymo kit were higher in some instances (Fig. S1b). Therefore, we used the Qiagen RNeasy micro kit for our study.

**Figure 1:**
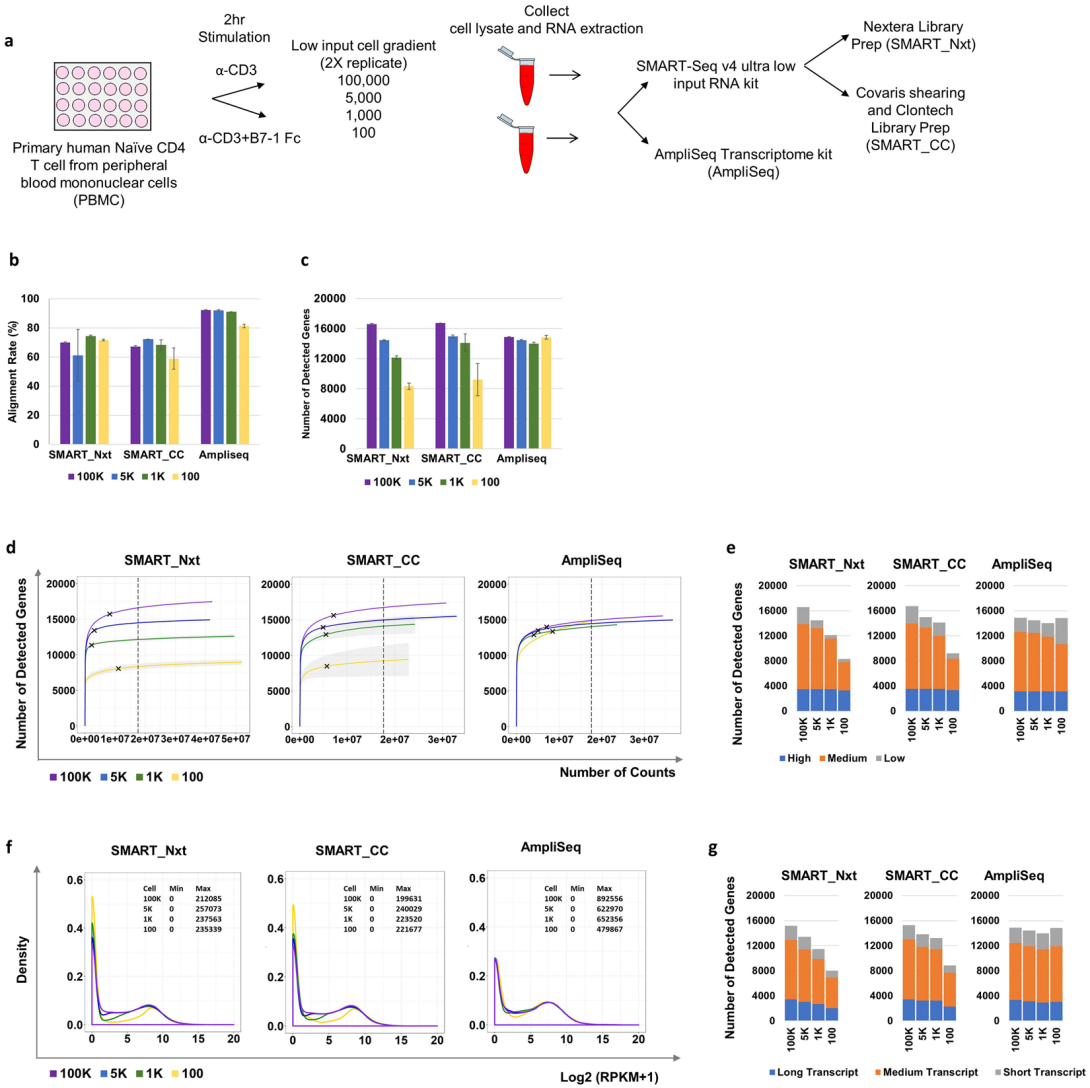
Number of detected genes decreased with reduced input in SMART technology, while it remained constant for AmpliSeq technology. (**a**) Experiment design for low-input RNA-Sequencing platform evaluation using stimulated primary human naïve CD4 T cells. Three protocols based on two technologies and four cell gradients were tested. (**b**) Alignment rates for samples at the four input cell gradients (100, 1 K, 5 K, 100 K) for the three protocols. Bar plot shows mean +/− standard deviation of the replicates. (**c**) Number of USCS genes detected (count > 0) for samples at the four input cell gradients (100, 1 K, 5 K, 100 K) for the three protocols. Bar plot shows mean +/− standard deviation of the replicates. (**d**) Collection curves showing the number of detected genes at different sequencing depths in SMART_Nxt (left), SMART_CC (middle), AmpliSeq (right). Solid lines indicate the mean and shading regions indicate standard deviation. Black crosses in each sample indicates the sequencing depth where 90% of the genes were detected. Vertical dashed black lines indicate sampled library size for downstream analysis. (**e**) Number of detected genes grouped into high, medium and low expressing genes. (**f**) Density plot showing the distribution of the log2 transformed RPKM values in each cell input in SMART_Nxt (left), SMART_CC (middle), AmpliSeq (right). Minimum and maximum RPKM values at each cell input were also listed on the upper right of the plot. (**g**) Number of detected genes grouped into short, medium and long transcripts. Samples from α-CD3+ B7-1 Fc treatment were used for all figures.

Primary human naïve CD4 T cells were treated with α-CD3 only or α-CD3 and B7-1 Fc, which stimulate T cell activation, for 2 hours. Next, the cells were collected and serially diluted to achieve 100 K, 5 K, 1 K and 100 cells. Finally, RNA was extracted and transcriptome library was generated with two different protocols: SMART-Seq and AmpliSeq. For SMART-Seq technology, two different library preparation kits were utilized: Nextera Library Preparation kit and Clontech Library Preparation kit (Fig. 1a). From this point forward, we will refer to the samples prepared with Nextera Library Preparation kit and sequenced with SMART-Seq as SMART_Nxt, the samples prepared with Clontech Library Preparation kit and sequenced with SMART-Seq as SMART_CC, and the samples prepared with AmpliSeq kit as AmpliSeq samples.

To evaluate the technical performance of the three protocols, we compared percentages of reads that aligned to reference genome (Human Genome hg19). For samples treated with α-CD3 and B7-1 Fc, the average alignment rates ranged between 59% to 74% for SMART_Nxt and SMART_CC. AmpliSeq mapping percentages across all cell gradients were between 81 to 92%. (Fig. 1b). Similar patterns were observed in samples treated with α-CD3 alone (Fig. S2a). PCR duplication is a common concern of ultra-low input methods, as it could introduce noise and bias the analysis. For SMART-Seq based protocols (SMART_Nxt and SMART_CC), the duplication rate can be computationally estimated as reads with the same start postion. Both protocols showed dramatic increase of PCR duplication rate with reduced input cell number, from <20% at 100 K cells to >60% (SMART_Nxt) or >90% (SMART_CC) at 100 cells. At lower cell input (1 K and 100), SMART_Nxt showed lower PCR duplication rate compared to SMART_CC (Fig. S2b). Since Ampliseq is by nature a PCR based technology, the duplication rate cannot be assessed with current version of the protocol. For gene annotation, we used the UCSC database. It contains total of 25369 genes, most of which are protein-coding. Among these genes, 20755 (81.8%) are included in the AmpliSeq detection panel. The AmpliSeq assay detects additional 47 genes (Fig. S2c). In order to compare the number of genes detected across protocols, an equal number of total aligned reads (~17million, which equals to the minimum number of reads across the samples, as depicted by the hashed line in Fig. 1d) was used per sample. SMART_Nxt and SMART_CC detected over 16,000 genes (count > 0) in the 100 K cell samples. This number reduced with decreased cell input, with only about 50% (8000) of the genes detected in the 100 cell samples. AmpliSeq detected ~15,000 genes in the 100 K cell samples, and notably comparable number of genes were detected even when the cell input decreased to 100 (Fig. 1c). To better understand the efficiency of gene detection with sequencing depth, for each sample we randomly extracted different number of counts and determined the number of detected genes. For majority of the samples, the gene detection was saturated (defined by 95% of the genes being detected) at ~10 million reads. Notably, for SMART_Nxt and SMART_CC the lower number of detected genes at low cell gradient could not be compensated by higher sequencing depth, while for AmpliSeq, the number of detected genes at different sequencing depth are comparable at different cell gradients (Fig. 1d). Furthermore, we grouped the genes by expression levels and for each platform, we used the 100 K sample as the reference. High expressing genes were defined as gene length adjusted count values (Reads per million per kb (RPKM)) >20 percentile, medium expressing genes as gene length adjusted count values between 20–80 percentile, and low expressing genes as gene length adjusted count values < 20 percentile of all detected genes. We observed that the detection of medium and low expressing genes were impacted as cell input decreased, but high expressing genes were not impacted for SMART_Nxt and SMART_CC. Detection rates were not affected by input level for AmpliSeq (Fig. 1e). This is consistent with the density distribution of gene expression levels. For SMART_Nxt and SMART_CC, with decreased cell input, we observed higher density of genes with RPKM of 0, and lower density of genes with low to medium RPKM values. For AmpliSeq, the density of genes with RPKM of 0 are comparable at diffenent cell inputs, although we still observed slightly lower density of genes with low to medium RPKM especially at 100 cells. However, for AmpliSeq, the maximum RPKM values were lower at low cell inputs (Fig. 1f). The effect of transcript length on gene detection was also explored. Here, we defined long transcripts as the ones with length >80 percentile, medium transcript as ones with length between 20–80 percentile, and short transcript as ones with length <20 percentile of the 20755 common gene targets in SMART_Nxt, SMART_CC and AmpliSeq. The transcript length had minimal impact on gene detection, as the ratio between the three group remains consistent in different cell inputs (Fig. 1g). Similar patterns were observed in samples treated with anti-CD3 alone (Fig. S2d–h). One advantage of the SMART-seq technology based protocols is the ability to detect non-coding genes and novel transcripts. Indeed, for both SMART_Nxt and SMART_CC, about 10% of the reads were mapped to non-UCSC annotated genes across diffenent cell inputs (Fig. S2i). We increased the sampled reads of these libraries by ~11% (1/0.9) and used a more comprehensive annotation file of hg19 (the Ensembl annotation) with total of 59573 genes. By doing this we detected ~10,000 additional genes at 100 K cells for both protocols, most of which were non-coding genes. Similar to the UCSC genes, the detection was dramatically decreased at lower cell inputs (Fig. S2j).

### High reproducibility between technical replicates above 1 K-cell input, but increased variability at 100-cell input

**Figure 2:**
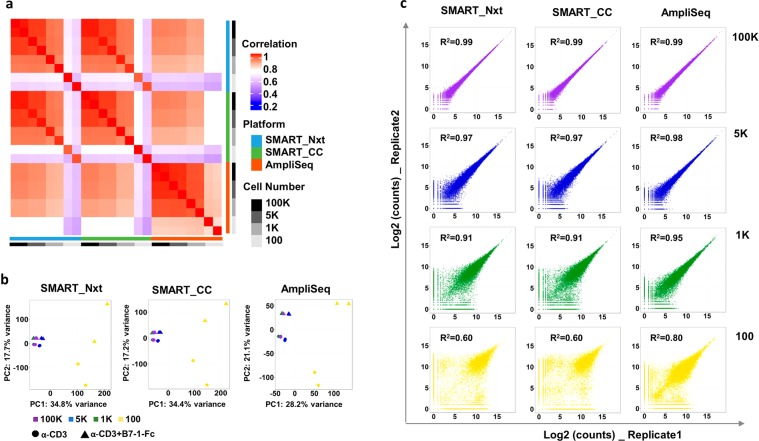
Consistency between technical replicates was high at cell input equal to or above 1 K, and there was increased variability at 100 cell input. (**a**) Heatmap showing Pearson correlation of log2 transformed count values (Blue indicates low correlation and red indicates high correlation). Samples from α-CD3 + B7-1 Fc treatment are shown. (**b**) PCA plot show global expression pattern for sample in each cell gradient in each platform. Samples from both α-CD3 and α-CD3 + B7-1 Fc treatment are shown. (**c**) Scatter plots show correlation between the two replicates for each cell gradient in each platform. R^2^ indicates coefficient of determination. Samples from α-CD3 + B7-1 Fc treatment are shown.

We examined the reproducibility of gene expression levels across protocols, technical replicates and cell inputs. Overall, high reproducibility was observed across protocols and input levels (R^2^ ≥ 0.8), except the 100 cell samples from SMART_Nxt and SMART_CC showing lower level of concordance (R^2^ < 0.8) with other samples (Fig. 2a). Consistent with this observation, the PCA plot showed tight clustering of technical replicates, and a clear separation of the samples by treatment condition (α-CD3 only or α-CD3 and B7-1 Fc). However, the samples with 100 cell input clustered separately than the other cell input groups and treatments, and also showed significant variability within replicates (Fig. 2b). A closer look at the correlation between replicates showed high correlation for the 100 K cell samples (R^2^ = 0.99 for all three protocols). As cell input decreased, the correlation also decreased, but were maintained at R^2^ > 0.9 up to the 1 K cell input samples. Replicate correlation dropped significantly with the 100 cell samples. The R^2^ values were 0.6 for SMART_Nxt and SMART_CC, and 0.8 for AmpliSeq (Fig. 2c). Similar correlation patterns were observed for the samples treated with α-CD3 alone (Fig. S3a,b).

### Cell input of 1 K or higher show high reproducibility across input levels; 100-cell input exhibits loss of reproducibility especially for low expressing genes

**Figure 3:**
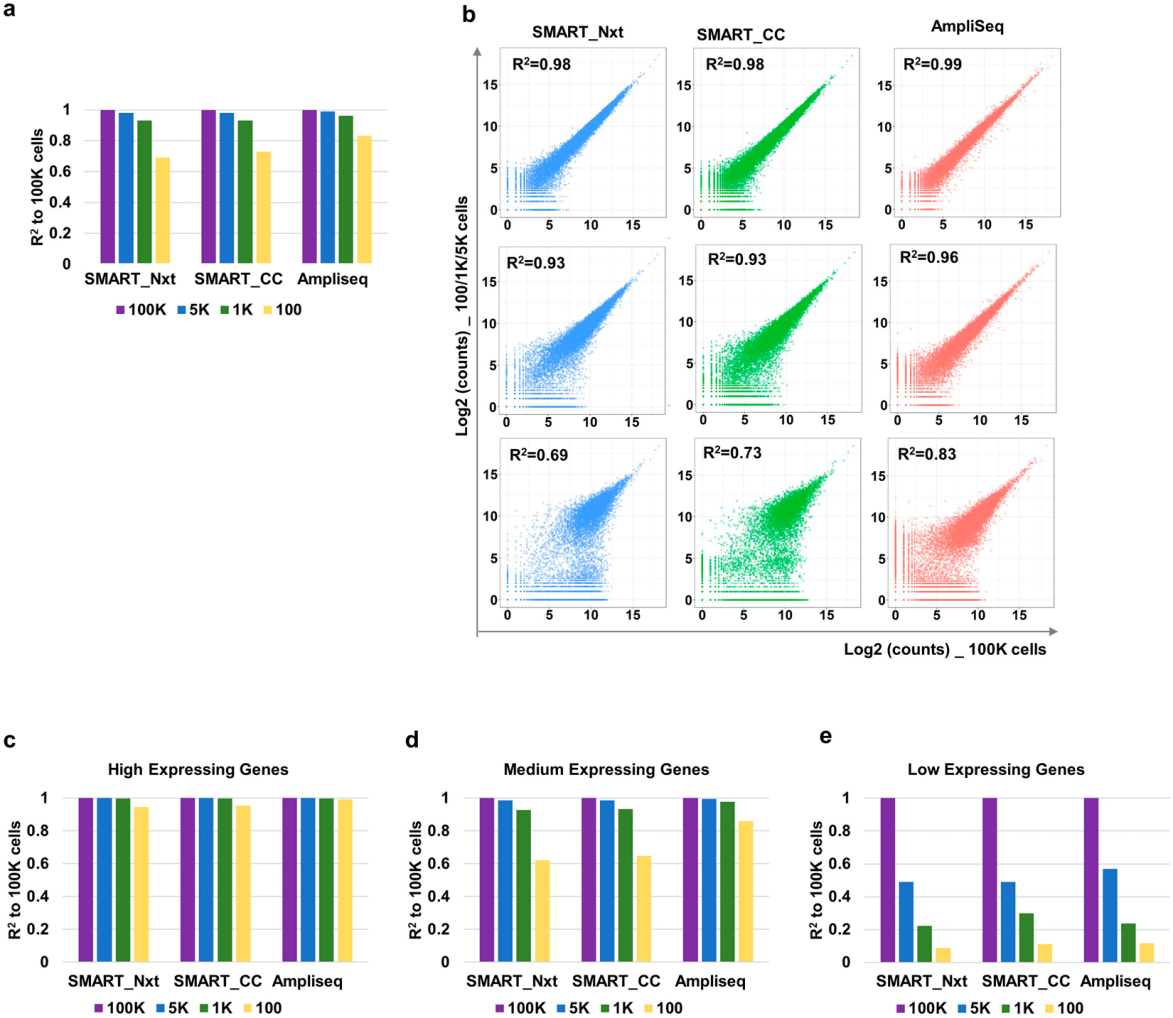
Consistency between different gradients was high at cell input equal to or above 1 K, and the greatest impact was observed on the loss of low expressing genes at 100 cell input. (**a**) Bar plots show correlation between 5 K, 1 K or 100 cell inputs and 100 K cells input for each platform. Replicate 1 of each set was used for the calculation. R^2^ indicates coefficient of determination. (**b**) Scatter plot show the correlation between samples from the 5 K (top), 1 K (middle), 100 (bottom) cells to samples from the 100 K cells for each platform. Replicate 1 was used for the calculation. (**c**–**e**) Bar plot show correlation between samples from the 5 K, 1 K or 100 cell input and 100 K cells for each platform. Panels are separated to represent (**c**) high, (**d**) medium and (**e**) low expressing genes. Replicate 1 was used for the calculation. R^2^ indicates coefficient of determination. In each figure, samples from α-CD3 + B7-1 Fc treatment were used.

We further evaluated whether expression profiles at low cell input still represent the cell population (100 K cells) by calculating the correlation coefficient between samples with lower inputs to the 100 K-cell samples. For all three protocols, samples with 5 K-cell and 1 K-cell samples showed high correlation with 100 K-cell samples (R^2^ > 0.9), while the correlation dropped to 0.7–0.8 with 100-cell samples. Notably, AmpliSeq samples showed better correlation across all cell inputs compared to those from SMART_Nxt and SMART_CC. Specifically, for the 100 cell inputs, AmpliSeq had the highest correlation compared to SMART_Nxt and SMART_CC (R^2^ = 0.83, 0.69 and 0.73 respectively) (Fig. 3a,b). To determine the impact of gene expression levels on the correlations, we again grouped the genes into high, medium and low expressing genes. High expressing genes showed highest correlations across all cell inputs (Fig. 3c). For medium expressing genes, the correlation of the 100 cell samples dropped to 0.6 for SMART_Nxt and SMART_CC while it remained above 0.8 for AmpliSeq (Fig. 3d). However, for low expressing genes, the correlation dropped significantly but to comparable levels for all three protocols (R^2^ < 0.2) (Fig. 3e). Again, similar results were observed in samples treated with α-CD3 (Fig. S4a–e).

### Three different protocols detected common differentially expressed genes; however platform-specific genes were also detected

**Figure 4:**
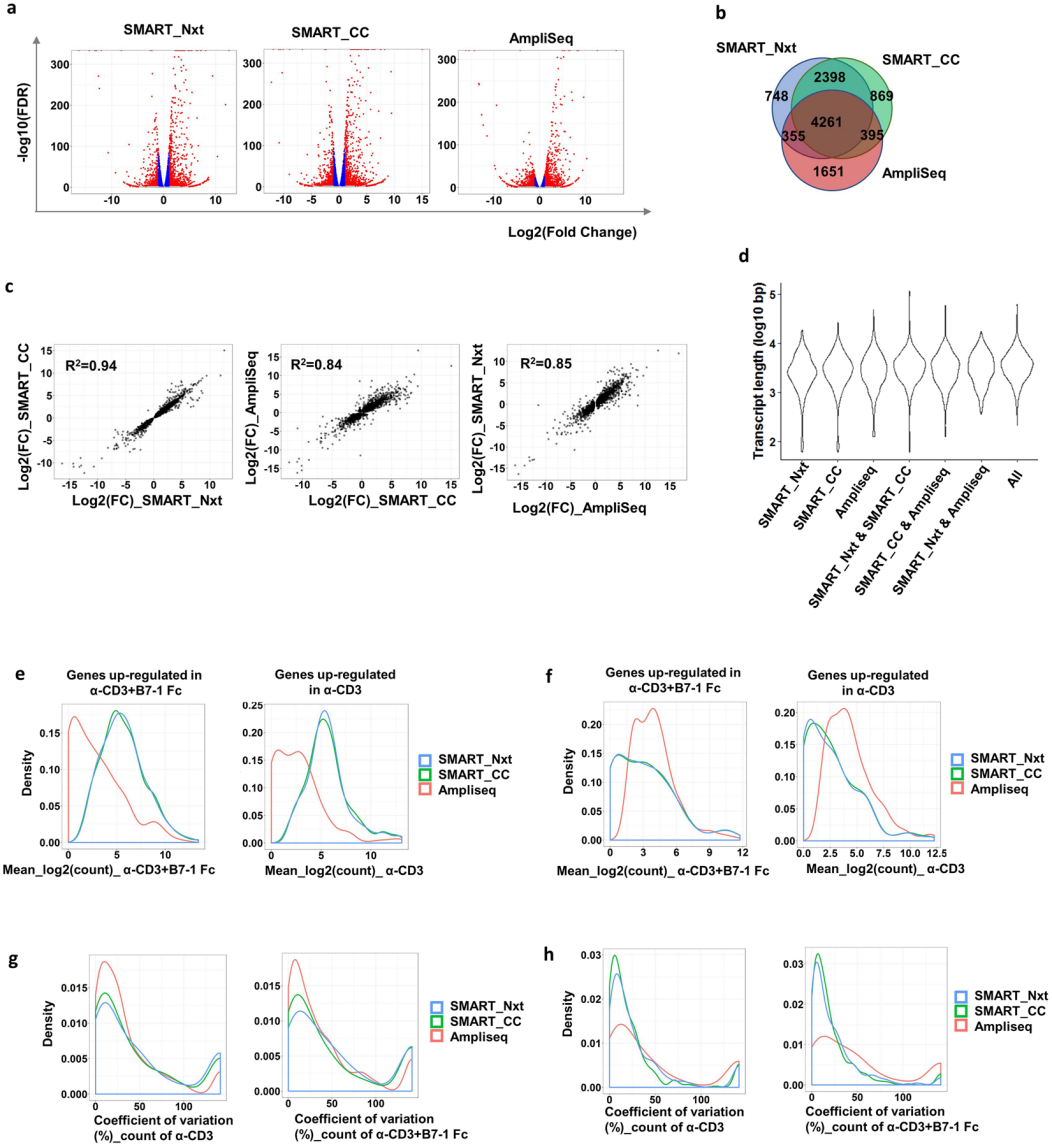
Three different platforms detected common differentially expressed genes; however platform specific detection was also observed. (**a**) Differential gene expression analysis for each platform. Red dots indicate genes with FDR < 0.05 and Fold change > = 2, blue dots indicate genes with FDR < 0.05 and Fold change < 2, grey dots indicate genes with FDR > 0.05. (**b**) Overlapping differentially expressed genes (FDR < 0.05) among platforms. (**c**) Scatterplots showing the log2 transformed fold change between each pair of the three platforms for the 4261 common DEGs. R^2^ indicates coefficient of determination. (**d**) Distribution of transcript lengths of the common and platform specific DEGs as in (**b**). (**e**) The distribution in expression of 2398 genes that were common for SMART_Nxt and SMART_CC (expression in α-CD3 + B7-1 Fc for genes up-regulated in α-CD3 + B7-1 Fc vs. α-CD3, and expression in α-CD3 for genes down-regulated in α-CD3 + B7-1 Fc vs. α-CD3). (**f**) Distribution of transcript lengths for AmpliSeq specific 1651genes. (**g**,**h**) The coefficient of variation (CV) between replicates for (**g**) SMART_Nxt and SMART_CC common DEGs and (**h**) AmpliSeq specific genes.

Differential gene expression is one of the most informative outcomes for a RNA-seq experiment, since it provides insights into distinct biological processes between treatments or state of cells and tissues (e.g. healthy vs. disease). Using 100 K cell samples as the reference, we examined the consistency between the three protocols in detecting differentially expressed genes (DEGs). Overall, 7762, 7923 and 6662 genes had significant differential expression between the two treatment groups (α-CD3 + B7-1 Fc vs α-CD3) in SMART_Nxt, SMART_CC and AmpliSeq, respectively (false discovery rate (FDR) < 0.05) (Figs 4a and S5a, Dateset 1). Among these, 4261 DEGs were detected by all three protocols. SMART_Nxt and SMART_CC showed higher similarity with 2398 common DEGs but not AmpliSeq. On the other hand, 1651 DEGs were detected by AmpliSeq but not the other two protocols (Fig. 4b). For the 4261 common DEGs that were detected by all three protocols, fold changes were consistent across protocols (R^2^ = 0.94 for SMART_Nxt and SMART_CC, R^2^ = 0.84 for AmpliSeq and SMART_CC; R^2^ = 0.85 for SMART_Nxt and AmpliSeq) (Fig. 4c). DEG fold changes were also consistent between SMART_Nxt and SMART_CC for the 2398 common DEGs of the two protocols (R^2^ = 0.91) (Fig. S5b). However, platform-specific DEGs showed low correlation between different protocols (data not shown). In order to understand the significance of platform-specific genes, we picked representative genes for qRT-PCR validation. Results are shown in Tables S1–6. By using qRT-PCR, we determined whether the genes were significantly upregulated or downregulated, and whether this matched the RNAseq data. In some cases, the differential expression pattern matched, and we considered those genes validated. In other cases, either there was no significant change or the direction of differential expression was opposite to what was observed in RNAseq data. In the latter cases, we considered those genes to be not validated. We were able to validate the following percentages: SMART_Nxt only ~72%, SMART_CC only ~50%, AmpliSeq only ~59%, SMART_Nxt and SMART_CC ~76%, SMART_Nxt and AmpliSeq ~57% and SMART_CC and Ampliseq ~66%. We also looked at the transcript length, which showed no difference on common or platform specific DEGs (Fig. 4d).

To better understand the detection of platform-specific DEGs, we first looked into the expression level of these genes. We plotted the distribution in expression of 2398 genes that were common for SMART_Nxt and SMART_CC (expression in α-CD3 + B7-1 Fc for genes up-regulated in α-CD3 + B7-1 Fc vs. α-CD3, and expression in α-CD3 for genes down-regulated in α-CD3 + B7-1 Fc vs. α-CD3). Indeed, the expression of these DEGs were higher in SMART_CC and SMART_Nxt than in AmpliSeq (Fig. 4e). On the other hand, the expression of 1651 AmpliSeq specific DEGs, were higher in AmpliSeq than SMART_Nxt and SMART_CC (Fig. 4f). Next, we studied the variation between technical replicates in platform specific DEGs, and plotted the coefficient of variation (CV) between replicates. SMART_Nxt and SMART_CC common DEGs indeed had lower CV in SMART_Nxt and SMART_CC, while the AmpliSeq specific DEGs had lower CV in AmpliSeq. (Fig. 4g,h).

### Number of differentially expressed genes decreased with reduced input, precision of detection stayed robust and AmpliSeq demonstrated greater sensitivity

**Figure 5:**
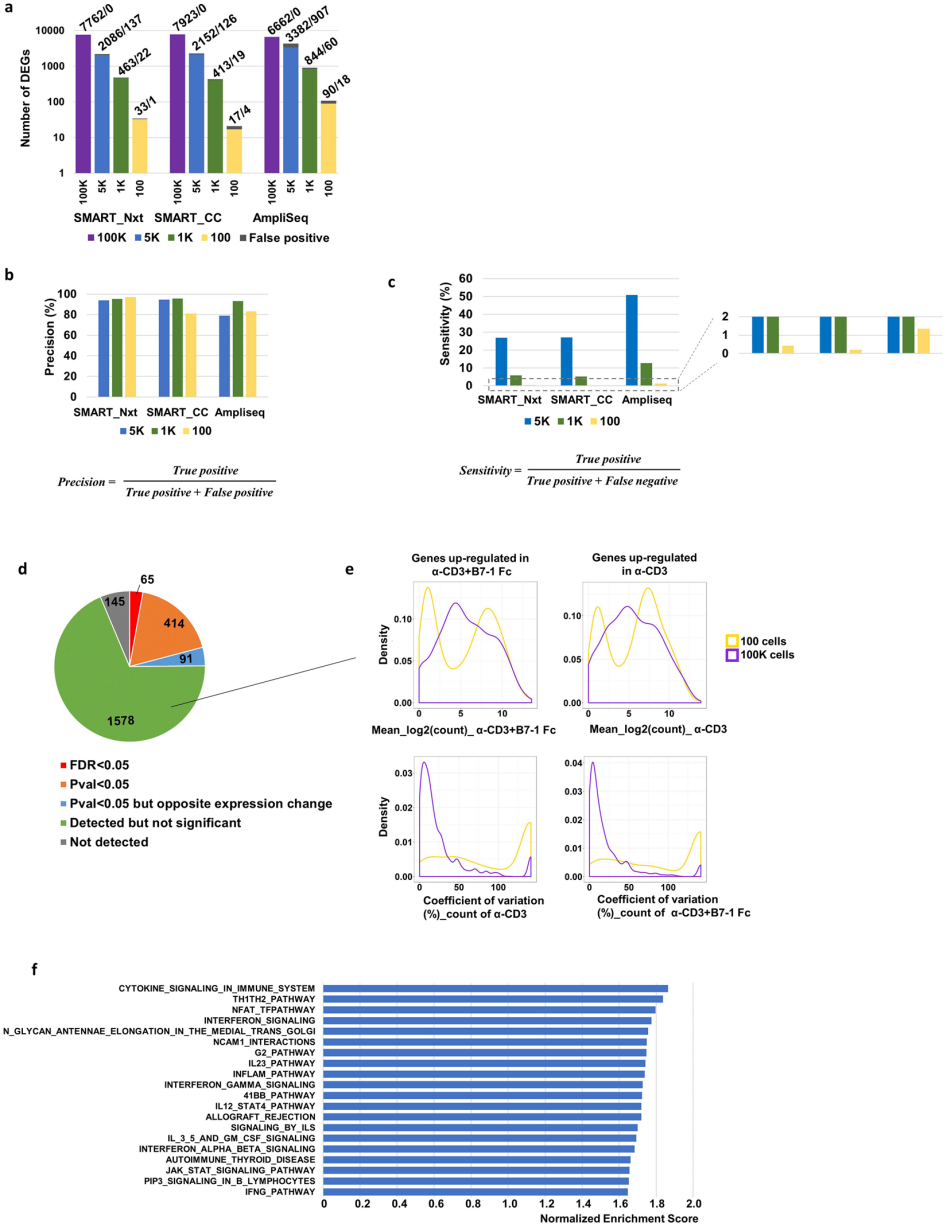
Number of differentially expressed genes decreased with reduced input, precision of detection stayed robust and AmpliSeq demonstrated greater sensitivity. (**a**) Number of DEGs between α-CD3 and α-CD3 + B7-1-Fc at 100 K, 5 K, 1 K and 100 cells input using Amplieq, SMART_Nxt and SMART_CC platforms (FDR < 0.05). Gray boxes indicate the DEGs not detected in 100 K cell samples (benchmark sample) but detected at low cell-number gradients (false positive hits). The absolute number of true positive and false positive detected genes were shown above each bar in the plot. (**b**) Precision for detecting DEGs at low input samples. (**c**) Sensitivity for detecting DEGs at low input samples. (**d**) Pie plot showing the differential expression analysis results for the DEGs detected in the 100 K samples (FDR < 0.05 and Fold change > 2) but not the 100 cell samples. (**e**) For the 1578 non-significant genes as in (**d**), density plots showing the mean log2 count and coefficient of variation between replicates for the 100 K cell samples and 100 cell samples. (**f**) Top 20 pathways enriched in α-CD3 + B7-1 Fc from the gene set enrichment analysis (GSEA) for the 100 cell sample. Bar plot shows the normalized enrichment score. All pathways passed the FDR < 0.25 as recommended by the GSEA team.

We also looked at the consistency of DEG detection at different cell inputs. At each cell input below 100 K, AmpliSeq detected higher number of DEGs compared to the other protocols. Input amount was correlated with number of DEGs. For example, AmpliSeq detected 6662 DEGs at the 100 K-cell input, 3382 (50.7%) of which were detected at the 5 K-cell input, 844 (12.6%) at the 1K-cell input, and only 90 (1.3%) at the 100-cell input. At each cell input, a small number of DEGs were also detected that were absent in the 100 K-cell samples (For AmpliSeq samples: 907 for the 5 K, 60 for the 1 K and 18 for the 100-cell inputs). A similar patterns were observed for SMART_Nxt and SMART_CC (Fig. 5a). Using the 100 K-cell samples as reference, we checked the precision (True positive/(True positive + False positive)) and sensitivity (True positive/(True positive + False negative)) at different cell inputs in each platform. This revealed that as input decreased, precision remained robust among all three protocols (Fig. 5b). On the other hand, AmpliSeq had better sensitivity compared to SMART_Nxt and SMART_CC at the 5 K-cell input (~50% versus ~25%). Sensitivity further dropped at 1 K and 100-cell inputs, with AmpliSeq showing higher sensitivity. (Fig. 5c). When using the 4261 common DEGs detected by all three platforms at 100 K cells (Fig. 4b) as reference, we again observed dramatic decrease of number of DEG detection with reduced cell input in all three protocols, with AmpliSeq showing higher sensitivity than the other two procols (Fig. S6a,b).

Out of the 6662 DEGs detected with 100 K input in AmpliSeq samples, 2293 genes showed >2 fold difference, and these genes were not detected to be significant at the 100-cell input. We then looked into the group comparison result of these genes in the 100-cell samples. Among these genes, 145 genes were not detected, 1578 genes were detected but did not achieve significance (FDR < 0.05 or P value < 0.05) in the group comparisons, while 570 genes achieved significance but showed expression change of <2 fold (note 91 genes showed opposite direction of expression change) (Fig. 5d). For the 1578 genes that did not achieve significance, a proportion of them showed lower expression values in the 100-cell samples compared to the 100 K cell samples. More importantly, majority of these genes showed high variation between the replicates compared to the 100 K-cell samples, reflected by a heavy right tail in the CV plot (Fig. 5e).

Since the 100-cell samples showed lower sensitivity in detecting DEGs, we performed gene set enrichment analysis (GSEA), which takes into consideration of overall expression change of gene sets, and therefore is more sensitive in capturing changes of certain biological processes or pathways. GSEA analysis against the canonical pathways of the Molecular Signatures Database detects 47 pathways enriched in α-CD3 + B7-1 Fc treatment (FDR < 0.25 as recommend cutoff by GSEA), with good representation of pathways associated with activation of T cells, such as cytokine signaling in immune system, Th1/Th2 pathway, NFAT pathway and Interferon signaling (Fig. 5f).

### Detection of established T cell activation markers was achieved at 1 K cell input and above, and AmpliSeq detected higher percentage of these genes at 100 cell input

**Figure 6:**
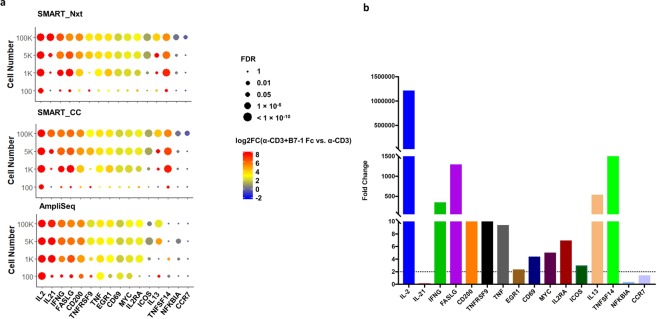
Detection of well-known T cell activation markers was achieved at 1 K cell input and above, and AmpliSeq detected higher percentage of these genes at 100 cell input. (**a**) Log2 fold change and FDR between α-CD3 and α-CD3 + B7-1-Fc for selected genes that were reported previously to be up-regulated at T cell activation. Log2 fold change is indicated by color and significance (FDR) is indicated by size of the points. (**b**) qRT-PCR analysis of selected genes, fold change in α-CD3 + B7-1 Fc group when compared to α-CD3 only is shown.

Previous studies have reported genes that are differentially expressed upon T cell activation (Chun Jimmie Ye *et al*., 2014). Here we looked into 15 of these genes (*IL2, IL21, IFNG, FASLG, CD200, TNFRSF9, TNF, EGR1, CD69, MYC, IL2RA, ICOS, IL13, TNFSF14, NFKBIA*, and *CCR7*) upon T cell activation, to examine how the detection of differential gene expression compared between different technologies. At higher inputs (100 K, 5 K and 1 K), the technologies performed similarly in detecting the fold change in these different genes. For the 100-cell samples, in Ampliseq, 7 (*CD200, CD69, EGR1, FASLG, IL2, MYC and TNF*) out of the 16 genes were significantly upregulated in α-CD3 and B7-1 Fc group versus α-CD3 only, while only 1 gene (*IL2*) was detected by SMART_Nxt and SMART_CC. However, for *TNFSF14*, which was detected to be differentially expressed by SMART_Nxt and SMART_CC, AmpliSeq was not able to detect any significant change even in the highest cell input (Fig. 6a). qRT-PCR analysis of the samples demonstrated that 12 out of 16 genes were differentially changed in α-CD3 and B7-1 Fc group versus α-CD3 only (Fold change > 2) (Fig. 6b). Interestingly, we did not detect significant upregulation of IL-21 with qRT-PCR, and this was unexpected. This may be due to the primer set that was used.

## Discussion

T cells are main players in adaptive immunity and have also been important targets in modulating different disease pathologies such as cancer and autoimmunity. Primary T cells can be limiting in numbers and are prime examples of cells with low RNA content. Given their importance, studying T cells at a whole-transcriptome level requires a platform that combines low amounts of RNA with reproducible and quantitative detection of subtle gene expression changes. Ability to detect gene expression levels quantitatively and reproducbly is the hallmark of a reliable gene expression analysis platform. Over the recent years, RNAseq has emerged as such a platform; however, standard protocols for RNAseq analysis requires several hundred nanograms of high-quality RNA. Hence, we evaluated the feasibility of studying gene expression changes underlying T cell activation using the low-input RNAseq methodology. We selected SMART-Seq, Ampliseq and two different library preparations for SMART-Seq for performing this evaluation based on published methods as well as product specifications provided by kit manufacturers. We compared these three methods to study differential gene expression in human T cells with α-CD3 and α-CD3 + B7-1 Fc activation.

As a first step, we compared three different RNA extraction and isolation methods, and demonstrated that Qiagen RNeasy micro kit performed best in extracting RNA from low cell numbers. In respect to gene detection rates, AmpliSeq had the best performance out of all three protocols. It is also notable that across protocols, increasing the sequencing depth beyond 10 million did not help in improving gene detection rates. This indicates that at very low input levels, the mRNA representation is lost at RNA extraction or library prep steps. While input levels did not affect the detection rate for high expressing genes across methods, we observed that Ampliseq was the only method that preserved the detection rates for medium and low expressing genes. This supports that the utilization of gene-specific oligonucleotides as primers during the library preparation to improve the detection of low expressors. SMART_Nxt and SMART_CC showed significant drop in detection rates at inputs below 100 K cells, with the highest impact on low expressing genes. Furthermore, detection rates were not impacted by transcript length for any method. Therefore, we demonstrate that AmpliSeq method had the best reproducible alignment rate and number of detected genes, regardless of input cell number. This is an important point to consider while deciding which RNAseq method to use for studies with low RNA input. For example, in instances of study designs where there are sample sets with low cell numbers as input, AmpliSeq may provide consistent performance in the above mentioned parameters across all samples in the experiment.

Technical reproducibility across input levels and methods were generally high for all methods, except at 100-cell input. At the 100-cell input, AmpliSeq outperformed other methods with a R^2^ = 0.80 compared to R^2^ = 0.6 for SMART_Nxt and SMART_CC. However, it is important to note that when we looked at reproducibility between different cell inputs, all methods performed sub-optimally at the 100 cell input, when compared to the 100 K-cell reference. This was especially evident when comparing medium and low expressing genes. This may suggest that the threshold for the detection of medium and low expressing genes reproducibly exists with 1 K-cell input and above. When using 100 cells as input, we need to keep in mind that the data would be mostly representative of high expressing genes, regardless of the technology employed.

In detecting DEGs, SMART_Nxt and SMART_CC were more concordant with each other than with Ampliseq, ostensibly due to the SMART technology that underlies both protocols. The discordance we observed between Ampliseq and the SMART methodologies in detecting certain specific DEGs could be either due to low representation of those genes in the library pool or high variability among replicates. It is to be noted that while precision remained high at all input levels, sensitivity of detecting DEG dropped significantly for all methods as the cell input decreased. Ampliseq had almost twice as much sensitivity at 5 K input, and slightly better sensitivity at 1 K and 100-cell inputs. Additionally, Ampliseq was also able to detect more biologically relevant gene expression changes compared to SMART_Nxt and SMART_CC at the 100 cell input level. However, at 100 cell input level, AmpliSeq detected only 1% of the DEGs compared to 100 K-cell input. Hence, when interpreting the data, additional approaches such as pathway enrichment would be helpful besides differential expression analysis. For SMART_Nxt and SMART_CC, the drop of sensitivity may at least be partialy explained by the dramatic increase of PCR duplication rate, which introduces noise to the data. Acually at 1 K and 100 cell inputs, SMART_Nxt performed slightly better than SMART_CC in DEG detection, consistent with lower PCR duplication rates of SMART_Nxt at these two cell input levels compared to SMART_CC. The PCR duplication rate is not assessible for AmpliSeq since the technology is in nature based on PCR amplification. Additional modifications, such as unique molecular index (UMI), may be needed for accurate evaluation.

It is also important to note that, although AmpliSeq technology showed superior performance in gene detection, replicate consistency and differential expression detection, it also has some disadvantages. First, since it is a technology based on PCR amplication, the genes that can be detected are limited to the pre-designed primer library, mostly the protein-coding genes with current version of the protocol. With the SMART-seq based protocols, it is possible to detect non-coding genes and novel transcripts. Second, the quality, including both specificity and sensitivity, of the primers is critical in the accurate detection of gene expression. One example of such a gene is TNFSF14, which is robustly detected using qPCR as well as SMARTseq-based protocols at high cell inputs. However, even at the 100 K-cell input, Ampliseq was not able to detect the expression of TNFSF14, probably due to sub-optimal primer designs. On the other hand, in SMARTseq-based protocols, the cDNA is sequenced and aligned across the gene in an unbiased way; providing an advantage for this technology.

Finally, droplet-based single cell RNA sequencing approaches are also popular in order to study heterogeneity and plasticity of cell populations. While single cell RNAseq has several advantages, bulk RNA sequencing still is preferable for reasons such as cost savings and ability to use frozen samples. The number of detected genes in single cell sequencing is usually lower compared to bulk sequencing due to high drop-out rate of low expressing genes. Additionally, recommended minimum number of cells to sequence is 500 (per 10X protocol) and the workflow of single-cell RNAseq requires approximately 20–30% more cells as input. Depending on the nature of scientific questions and in circumstances in which only 100–500 cells are available, bulk RNA sequencing may be the preferable method.

While it is important to investigate the three different protocols in regard to different technical criteria, we also evaluated the biological meaning of the data acquired in this study. An expected T cell response against a pathogen or tumor cells requires an intricate response involving many genes. Stimulation of T cells with signal 1 (α-CD3) and signal 2 (B7-1 Fc) further results in upregulation of activation markers (i.e. *CD69* and *IL2RA*) and other co-stimulatory and co-inhibitory receptors (i.e. I*COS, TNFRSF9, TNFSF14* and *CD200*), in order to optimize the effector response. *ICOS* (CD278) is a co-stimulatory molecule that plays an important role in tumor immunity. It has been demonstrated that the efficacy of Ipilimumab (anti CTLA4) was partly due the recruitment of ICOS+ T cells into the tumor; and that in mice lacking ICOS, anti-CTLA4 therapy was much less effective^14^. CD200, on the other hand is a co-inhibitory receptor, and along with others such as PD-1, is upregulated as a self-check mechanism of immunity. Interestingly, *ICOS* was detected at 100 K and 5 K inputs with SMART_Nxt and SMART_CC, and the detection was not as robust with AmpliSeq technology. This may be due to poor primer design that was discussed previously. On the other hand, AmpliSeq technology was able to detect *CD200* differential gene expression even at 10-cell input.

One of the hallmarks of T cell activation is Interleukin 2 (IL-2) secretion. When T cells are activated through their TCR and co-stimulated through *CD28*, *IL2* mRNA is rapidly upregulated (data not shown). In general, there is more than 1000-fold increase of *IL-2* mRNA at 2 hr after α-CD3 and B7-1 Fc stimulation, and this fold change gradually decreases to baseline in the following hours. In this study, while IL-2 was detected by all technologies, AmpliSeq detection level at 100 cells was equivalent to 100 K cell input, demonstrating superior sensitivity. Another marker that was detected equally at 100 K and 100 cell input was Tumor Necrosis Factor (*TNF*), which also is an important inflammatory marker in T cell activation.

Activated T cells secrete many different cytokines, and depending on the milieu, they will skew into T helper 1, T helper 2, T helper 17 or T regulatory subtypes. One important T helper 1 cell effector cytokine is Interferon-gamma (IFNG). This cytokine is especially important in mounting an effector response against cancer cells and viral antigens^15^. In certain cases, only a very small number of tumor-infiltrating cells or virus-specific T cells can be isolated from patients, and it would be important to know which method would be effective in detecting this cytokine. AmpliSeq showed increased detection sensitivity at 100-cell input when compared with SMART technology. Another cytokine that is CD4 T cell-specific and important in cancer setting is Interleukin 21 (IL-21). IL-21 is upregulated in Th2 and Th17 subtypes and it has been demonstrated to increase CD8 T cell cytotoxic response. CD8 T cells primed with IL-21 also do not require CD4 T cell priming for cytotoxic killing responses^16,17^. Even at lower cell inputs, Amliseq was able to detect the mRNA of this cytokine.

*CCR7* (CD194) is important in homing of T cells into the secondary lymphoid organs, and usually is downregulated upon T cell activation. It has been demonstrated that this reduction in CCR7 is paralleled by upregulation of CXCR5 in the tonsil, and that the expression of these markers would dictate where the cells would locate within the organ (CCR7: resting vs CXCR5: activated)^18^. CCR7 expression is also different in different tissues and it may be a predictor of the number of Ag-engaging T cells, so detection of this marker would be important. Interestingly, Nextera and CC detected downregulation of *CCR7* at 100 K input, and not in any other cell gradients. In the case of AmpliSeq, downregulation was not detected at any input, and this may be addressed by better primer design.

Engagement of receptors on the surface of the T cells translate into intricate series of events at the intracellular level. Many transcriptional regulators are activated in order to orchestrate inflammatory gene expression. The earliest marker of CD28 engagement by B7-1 Fc is the family of early growth factor proteins. EGR1 regulates both Th1 and Th2 cytokine expression by modulating other transcription factor expression such as T-bet^19^. In a different study, it was demonstrated that EGR1 along with NFAT and NFKB binds to the IL-4 promoter in activated T cells and modulate the expression of this cytokine. In our study, we were able to detect *EGR1*, *MYC* and *NFKB* with all the technologies tested; however, as in other instances, Ampliseq method was more sensitive in detecting these genes at the 100-cell input.

In conclusion, at cell input of 100 K, 5 K and 1 K, all three methods are comparable in most of the criteria tested. AmpliSeq technology demonstrated some advantage over SMART technology: (a) the number of detected genes stayed constant regardless of input and even with low expressing genes, and (b) there was greater sensitivity in differential gene expression detection, especially at 5 K input. Furthermore, at 100-cell input, it may be better to employ AmpliSeq targeted technology due to increased detection of differential gene expression and comparable amount of sequencing data available regardless of input. However, it has to be noted that, even with the best of these methods, there is significant loss of information at the medium and low exressing genes at the 100-cell level. When targeting low expressing genes at 100-cell input or lower, targeted approaches like qRT-PCR may be best suited. For cell types that are similar to T cells in terms of RNA content, we expect that these three technologies will behave similarly at cell numbers 1000 and above; however this study clearly demonstrates the platform-specific outcomes of a RNA sequencing study.

## Methods

### T cell isolation and *in-vitro* activation

All experiments using human blood were approved and carried out in accordance with Advarra institutional review board (IRB). All experimental protocols were approved by MedImmune Blood Donor program. Written consent was obtained from adult healthy volunteers who are employees of MedImmune or Astrazeneca, and anonymized for research purposes. Donors positive for HIV infection, Hepatitis B or C virus, Human T-lymphotropic virus or syphilis are excluded from the donor program. Naïve CD4 T cells were isolated from the PBMC of donors with Easy Sep negative selection kit (Miltenyi # 19155). The cells were then plated in 24-well plates (Sigma # CLS3524) that were previously coated with α-CD3 (BioLegend # 317326) alone or a-CD3 and B7.1-Fc (R&D Biosystems # 140-B1). The plates were coated at 37 °C for 2 hours, and washed with PBS extensively prior to the addition of cells. 2.5 × 10^5^ cells were added per well and incubated for 2 hours at 37 °C and collected for RNA isolation. QIAshredder (Qiagen # 79654) was used according to manufacturer’s instructions to obtain T cell lysates.

### RNA Extraction for comparing different RNA extraction kits

T cells were collected in 200 µl of RLT Buffer with Beta-mercaptoethanol and serially diluted to achieve 100000, 5000, 1000 and 100 cells per sample. Total RNA from the cells was extracted using Qiagen’s RNeasy Micro Kit (Catalog #74004) according to the manufacturer’s guidelines with DNAse treatment and eluted in 18 µl of RNase-free water. To assess the quality and quantity of the RNA, the samples were analyzed on the Agilent 4200 Tape Station using the Agilent High Sensitivity RNA Screen Tape.

### cDNA Synthesis of Ultra-low Input RNA for Illumina Sequencing Protocols

The SMART-Seq v4 Ultra-low Input RNA Kit for Sequencing (Takara #634888) was used to generate high-quality full-length cDNA. In summary, the RNA was primed by the 3′ SMART-Seq CDS Primer II A for first-strand cDNA synthesis and used the SMART-Seq v4 Oligonucleotide for template switching at the 5′ end of the transcript. First-strand synthesis was directly followed by cDNA amplification by LD-PCR, which was carried out based on input amount of total RNA recommendations listed in protocol: 8 PCR cycles for 100 K samples and 11 cycles for remaining samples. The PCR-amplified cDNA were then purified and validated on the Agilent High Sensitivity DNA chip using the Agilent 2100 Bioanalyzer. The final cDNA product was aliquoted into two 5ul reactions, one for Clontech Low Input library preparation for Illumina whole transcriptome sequencing on the NextSeq500, and the other for Nextera Low Input library preparation for whole transcriptome sequencing on the HiSeq2000.

### Clontech Low Input Library Preparation for Illumina Whole Transcriptome Sequencing on NextSeq500

The cDNA was sheared using the Covaris M220 focused-ultra sonicator (SonoLab version 7.1) with run settings for desired target size between 200–500 bp. Library preparation was then performed following manufacturer’s instructions for Low Input Library Prep Kit v2 (Clontech # 634899). In brief, the sheared cDNA first underwent Template Preparation and Library Synthesis followed by Library Amplification where Illumina compatible dual index barcoded sequences were added to each sample. The recommended number of amplification cycles for different amounts according to the Low Input Library Prep Kit v2 user manual was used as a guideline for optimal amplification: 6 PCR cycles for 100 K/5 K samples and 10 cycles for 1 K/100 samples. The purified amplified libraries were then validated by Agilent High Sensitivity DNA chip on Agilent 2100 Bioanalyzer and quantitated via qPCR using KAPA Library Quantification Kit (KAPA Biosystems) according to manufacturer’s instructions. Libraries were then normalized to 4 nM each and pooled for a total of five Clontech libraries per pool and sequenced on Illumina’s NextSeq500 sequencer with the following run parameters: Paired-End/Dual-Indexed 2 × 75 bp reads.

### Nextera Low Input Library Preparation for Illumina Whole Transcriptome Sequencing on HiSeq2000

The cDNA was normalized to the modified recommended input amount of 100–150 pg based on Clontech’s SMART-Seq v4 Ultra-low RNA protocol. The samples were then tagmented according to Nextera XT DNA Library Prep Protocol (Illumina # FC-131-1024). During this step, the cDNA was simultaneously fragmented and tagged with sequencing adapters in a single enzymatic reaction. The fragmented cDNA was amplified for 12 PCR cycles and barcoded with Illumina Nextera XT DNA dual-index sequences. The amplified libraries did not undergo bead-based normalization as final step of protocol, and instead were purified using Ampure XP beads (Beckman Coulter). The purified amplified libraries were then validated by Agilent High Sensitivity DNA chip on Agilent 2100 Bioanalyzer and quantitated via qPCR using KAPA Library Quantification Kit (KAPA Biosystems) according to manufacturer’s instructions. Libraries were normalized to 4 nM each and pooled for a total of 4 Nextera libraries per pool, and sequenced on Illumina’s HiSeq2000 sequencer (1 pool per lane) with the following run parameters:Paired-End/Dual-Indexed 2 × 75 bp reads.

### Ampliseq Library Preparation for Targeted Transcriptome Sequencing

For each sample, 3.5 ul of RNA was used for input into the Ion Ampliseq Transcriptome Human Gene Expression Kit (Thermo Fisher # A26325). Due to variations in RNA concentrations, this resulted in an input of 0.69 ng to 10.61 ng for each Ampliseq reaction. For target amplification, samples with 8–10 ng RNA input underwent 12 PCR cycles while samples with less than 8 ng RNA input underwent 16 PCR cycles. Barcoded Ampliseq libraries were eluted in 50ul low TE and quantified using the Ion Library TaqMan Quantification Kit (Thermo Fisher #4468802). Libraries were normalized to 25pM for templating on the Ion Chef (Thermo Fisher). Different replicates of the same original diluted cell pellets were templated and sequenced on the same Ion Proton PI chip (Thermo Fisher) for a total of 8 Proton runs. Three libraries with less than 20 million mapped reads per library were re-templated and re-sequenced in order to obtain adequate reads.

### Bioinformatics analysis

For all samples, sequencing depth were designed to be two times of the recommended depth (i.e. 20 M for Ampliseq and 40 M for Clontech and Nextera). For Clontech and Nextera samples, Fastq files were quality checked using FastQC and further aligned to human genome (hg19) using Hisat2 (2.0.2-beta)^20^. PCR duplication rates were determined using Picard (http://broadinstitute.github.io/picard/), after the bam files were normalized to the size of the smallest sample. Gene count tables were generated using HTSeq (version 0.5.3p9)^21^. For Ampliseq samples, alignment and gene expression count were performed using Ion Torrent ampliSeqRNA Plugin v0.5.4.0 (Thermo Fisher) using hg19 genome.The count tables were further normalized to count per million (CPM) using edgeR (R Bioconductor)^22^. Differential expression gene analysis was performed using edgeR. False discovery rate (FDR) value was calculated based on the p-value using Benjamini–Hochberg procedure and genes with FDR < 0.05 were considered to be significant. Pathway analysis was performed using Gene set enrichment anlays (GSEA)^23^. Heatmap visualization was built using pheatmap (R). Statistical analysis was performed using R. Graphs were generated using R or Office Excel. All RNA sequencing data have been uploaded to Gene Expression Omnibus (GEO) with the accession number GSE130882 (https://www.ncbi.nlm.nih.gov/geo/query/acc.cgi?acc=GSE130882).

## Supplementary information

Dataset 1

Supplemental Information
