# Supplementary material for: Evaluation of ultra-low input RNA sequencing for the study of human T cell transcriptome
Source: Sci Rep. 2019 Jun 11;9:8445. doi: 10.1038/s41598-019-44902-z (PMC6559993; doi:10.1038/s41598-019-44902-z)
Supplement: Supplementary file 2 — Supplemental Information [file 41598_2019_44902_MOESM2_ESM.pdf]

**Evaluation of ultra-low input RNA sequencing for the study of human T cell transcriptome**

Jingya Wang[+1*], Sadiye Amcaoglu Rieder[+2], Jincheng Wu[$3], Susana Hayes[4], Rebecca Anne Halpin[4], Melissa de los Reyes[4], Yashaswi Shrestha[4], Roland Kolbeck[5] and Rajiv Raja[4]

+ contributed equally

* Correspondence to wangjin@medimmune.com

1 Translational Science and Experimental Medicine, Early Respiratory, Inflammation and Autoimmunity, R&D Biopharmaceuticals, AstraZeneca, Gaithersburg, USA

2 Biosciences, Early Respiratory, Inflammation and Autoimmunity, R&D Biopharmaceuticals, AstraZeneca, Gaithersburg, USA

3 Research Bioinformatics, MedImmune AstraZeneca, Gaithersburg, USA

4 Translational Medicine, Early Oncology, R&D Oncology, AstraZeneca, Gaithersburg, USA

5 Respiratory, Autoimmune and Inflammation, MedImmune AstraZeneca, Gaithersburg, USA

[$] Current address: Novartis Institutes for BioMedical Research

**Figure S1**

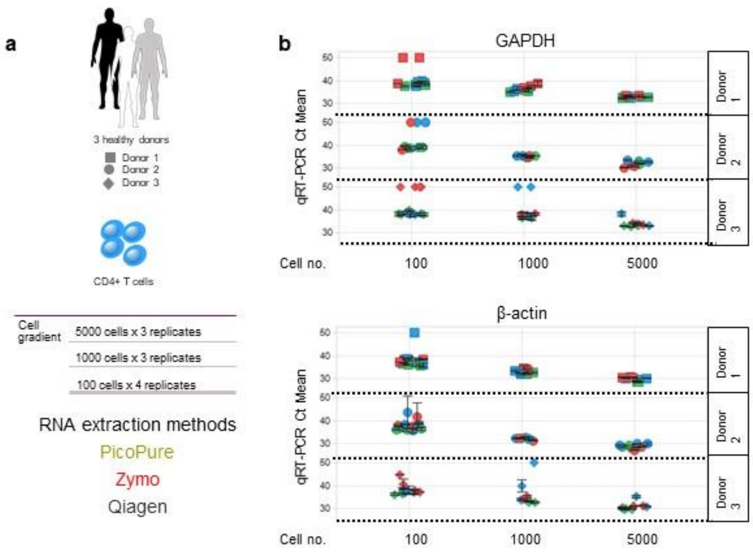
a
3 healthy donors
Donor 1
Donor 2
Donor 3
CD4+ T cells
Cell gradient
5000 cells x 3 replicates
1000 cells x 3 replicates
100 cells x 4 replicates
RNA extraction methods
PicoPure
Zymo
Qiagen
b
GAPDH
β-actin
qRT-PCR Ct Mean
Donor 1
Donor 2
Donor 3
Cell no.
100
1000
5000

**Figure S2**

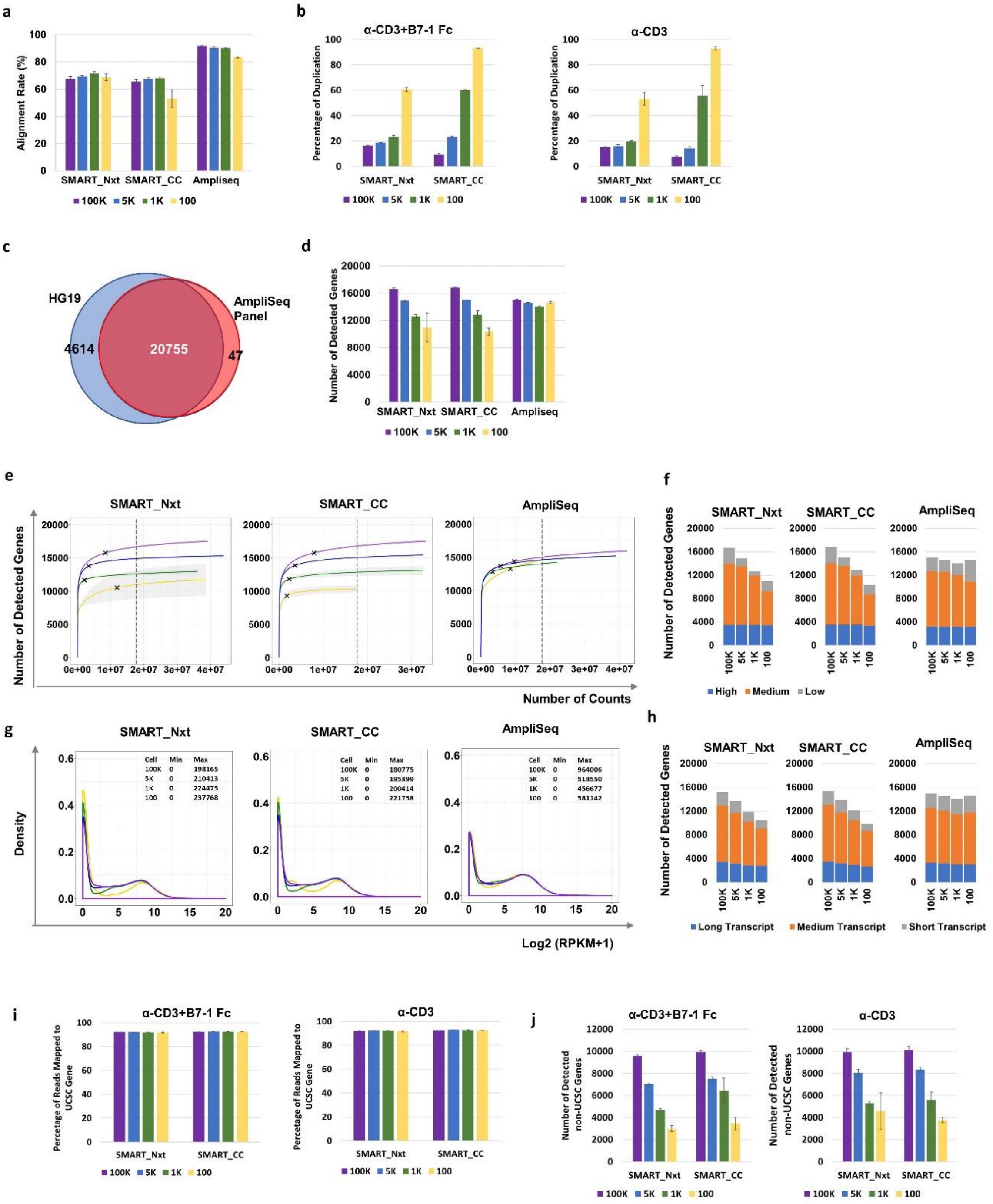
a
Alignment Rate (%)
SMART_Nxt
SMART_CC
Ampliseq
100K 5K 1K 100
b
α-CD3+B7-1 Fc
α-CD3
Percentage of Duplication
c
HG19
AmpliSeq Panel
4614
20755
47
d
Number of Detected Genes
e
SMART_Nxt
SMART_CC
AmpliSeq
Number of Detected Genes
Number of Counts
f
High
Medium
Low
g
Density
Log2 (RPKM+1)
h
Long Transcript
Medium Transcript
Short Transcript
i
Percetage of Reads Mapped to UCSC Gene
j
Number of Detected non-UCSC Genes

**Figure S3**

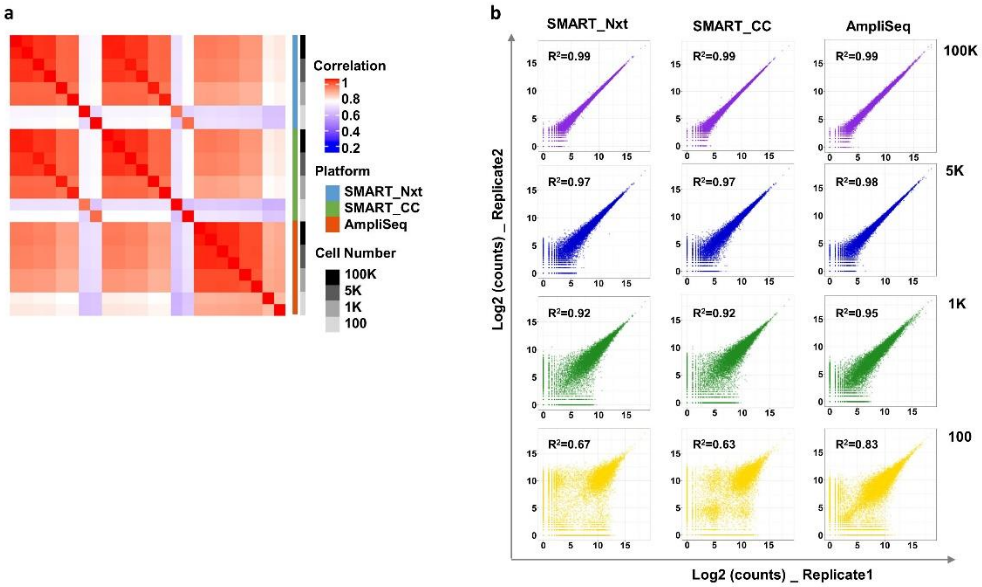

a
Correlation
1
0.8
0.6
0.4
0.2
Platform
SMART_Nxt
SMART_CC
AmpliSeq
Cell Number
100K
5K
1K
100
b
SMART_Nxt
SMART_CC
AmpliSeq
100K
5K
1K
100
R²=0.99
R²=0.99
R²=0.99
R²=0.97
R²=0.97
R²=0.98
R²=0.92
R²=0.92
R²=0.95
R²=0.67
R²=0.63
R²=0.83
Log2 (counts) _ Replicate2
Log2 (counts) _ Replicate1

**Figure S4**

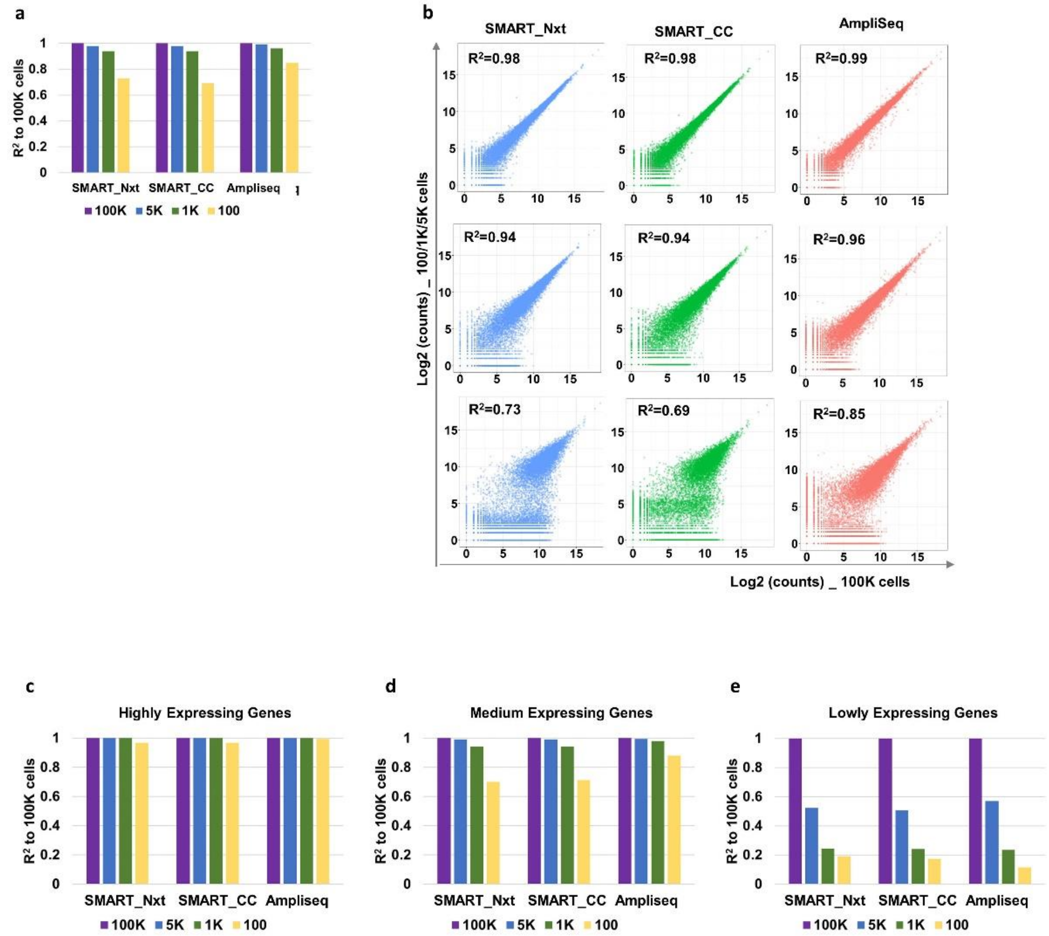
a
R² to 100K cells
1
0.8
0.6
0.4
0.2
0
SMART_Nxt
SMART_CC
Ampliseq
100K
5K
1K
100
b
SMART_Nxt
SMART_CC
AmpliSeq
R²=0.98
R²=0.98
R²=0.99
R²=0.94
R²=0.94
R²=0.96
R²=0.73
R²=0.69
R²=0.85
Log2 (counts) _ 100/1K/5K cells
Log2 (counts) _ 100K cells
c
Highly Expressing Genes
d
Medium Expressing Genes
e
Lowly Expressing Genes
R² to 100K cells
SMART_Nxt
SMART_CC
Ampliseq
100K
5K
1K
100

## Figure S5

**a**

| SMART_Nxt | | | | SMART_CC | | | | AmpliSeq | | | |
|---|---|---|---|---|---|---|---|---|---|---|---|
| 7762 | 94% | 95% | 97% | 86% | 94% | 95% | 100% | 69% | 80% | 88% | 85% |
| 2086 | 2223 | 81% | 44% | 94% | 88% | 84% | 38% | 76% | 69% | 60% | 68% |
| 463 | 395 | 485 | 41% | 96% | 80% | 91% | 24% | 84% | 81% | 50% | 55% |
| 33 | 15 | 14 | 34 | 91% | 44% | 38% | 76% | 85% | 71% | 44% | 26% |
| 6659 | 2092 | 465 | 31 | 7923 | 94% | 96% | 81% | 70% | 80% | 89% | 83% |
| 2144 | 1955 | 390 | 15 | 2152 | 2278 | 84% | 38% | 76% | 70% | 61% | 70% |
| 410 | 364 | 393 | 13 | 413 | 361 | 432 | 24% | 85% | 82% | 54% | 52% |
| 21 | 8 | 5 | 16 | 17 | 8 | 5 | 21 | 71% | 52% | 29% | 19% |
| 4616 | 1680 | 408 | 29 | 4656 | 1729 | 369 | 15 | 6662 | 79% | 93% | 83% |
| 3414 | 1539 | 392 | 24 | 3445 | 1589 | 354 | 11 | 3382 | 4289 | 89% | 80% |
| 796 | 544 | 243 | 15 | 808 | 547 | 235 | 6 | 844 | 806 | 904 | 63% |
| 92 | 73 | 59 | 9 | 90 | 76 | 56 | 4 | 90 | 86 | 68 | 108 |

**Platform**
- SMART_Nxt
- SMART_CC
- Ampliseq

**Cell Number**
- 100K
- 5K
- 1K
- 100

**b**

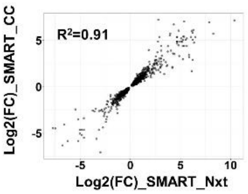

**Figure S6**

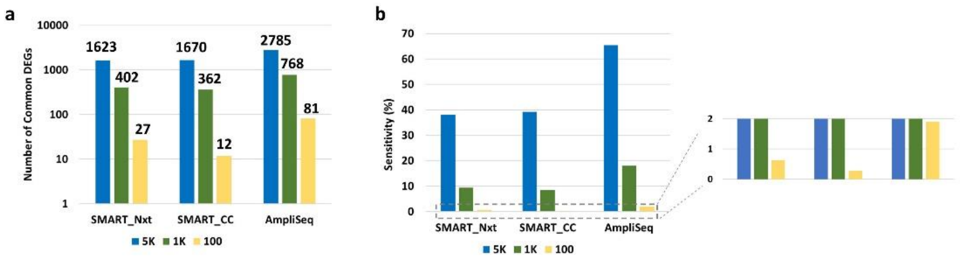
a
10000
1000
100
10
1
Number of Common DEGs
1623
402
27
1670
362
12
2785
768
81
SMART_Nxt
SMART_CC
AmpliSeq
5K
1K
100
b
70
60
50
40
30
20
10
0
Sensitivity (%)
SMART_Nxt
SMART_CC
AmpliSeq
5K
1K
100
2
1
0

## Figure Legend

**Figure S1. Comparing RNA extraction methods with different cell inputs**

(a) RNA extraction protocol comparison. CD4+ T cells were extracted from three healthy donors. Cells gradients of 100 to 5,000 were used with 2-4 replicates each per RNA extraction method. (b) qRT-PCR showed consistent amplification of 1000 and 5000 cell input. At 100 cells input, the ct mean values for Qiagen remained consistent among replicates, across donors for both housekeeping genes, GAPDH and β-actin. Zymo extraction could not detect GAPDH in the majority of samples, while multiple replicates failed to amplify GAPDH with PicoPure extracted RNA.

**Figure S2. Number of detected genes decreased with reduced input in SMART technology, while it remained constant for AmpliSeq technology**

(a) Alignment rates for samples at the four input cell gradients (100, 1K, 5K, 100K) for the three technologies. Bar plot shows mean +/- standard deviation of the replicates. (b). PCR duplication rates for samples at the four input cell gradients (100, 1K, 5K, 100K) for SMART_Nxt and SMART_CC from samples of α-CD3+B7-1 Fc (left) or α-CD3 (right) treatment. Bar plot shows mean +/- standard deviation of the replicates. (c) Venn diagram showing overlap of genes detected in the AmpliSeq panel and those annotated in UCSC hg19 database. (d) Number of gene detection (count>0) for samples at the four input cell gradients (100, 1K, 5K, 100K) for the three technologies. Bar plot shows the mean mapping percentage +/- standard deviation of the two replicates. (e) Collection curves showing the number of detected genes at different sequencing depths. Solid lines indicate the mean and shading regions indicate standard deviation. Black crossings indicate, for each sample, the sequencing depth where 90% of the

genes were detected. Dashed black lines indicate sampled library size for downstream analysis. (f) Number of detected genes, grouped into high, medium and low expressing genes. (g) Density plot showing the distribution of the log2 transformed RPKM values in each cell input in SMART_Nxt (left), SMART_CC (middle), AmpliSeq (right). Minimum and maximum RPKM values at each cell input were also listed on the upper right of the plot. (h) Number of detected genes, grouped into short, medium and long transcripts. (i) Percentage of the reads that mapped to UCSC genes at the four input cell gradients (100, 1K, 5K, 100K) for SMART_Nxt and SMART_CC from samples of α-CD3+B7-1 Fc (left) or α-CD3 (right) treatment. Bar plot shows mean +/- standard deviation of the two replicates. (j) Number of detected non-UCSC genes at the four input cell gradients (100, 1K, 5K, 100K) for SMART_Nxt and SMART_CC from samples of α-CD3+B7-1 Fc (left) or α-CD3 (right) treatment. Bar plot shows mean +/- standard deviation of the two replicates. For Figure (a, d-h), samples from α-CD3 treatment were used.

**Figure S3**. **Consistency between technical replicates was high at cell input equal to or above 1K, and there was increased variability at 100 cell input**

(a) Heatmap showing the correlation of log2 transformed count values (Blue indicates low correlation and red indicates high correlation). (b) Scatter plots showing the correlation between the two replicates for each cell gradient in each platform. $R^2$ indicates coefficient of determination. Samples from α-CD3 treatment were used.

**Figure S4. Consistency between different gradients wass high at cell input equal to or above 1K, and the greatest impact was observed on the loss of low expressing genes at 100 cell input**

(a) Bar plot showing the correlation between samples from the 5K, 1K and 100 cell input to samples from the 100K cells for each platform. Replicate 1 was used for the calculation. $R^2$ indicates coefficient of determination. (b) Scatter plot showing the correlation between samples from the 5K (top), 1K (middle), 100 (bottom) cells to samples from the 100K cells for each platform. Replicate 1 was used for the calculation. R2 indicates coefficient of determination. (c) Bar plot showing the correlation between samples from the 5K, 1K and 100 cell gradient to samples from the 100K cells for each platform, splitting into high, (d) medium and (e) low expressing genes. Replicate 1 was used for the calculation. $R^2$ indicates coefficient of determination. In each figure, samples from α-CD3 treatment were used.

**Figure S5. Three different platforms detected common differentially expressed genes; however platform specific detection was also observed**

(a) Percentage (upper triangle) and the absolute number (lower triangle) of differentially expressed genes that overlap between each pair of comparisons. The percentages were calculated as the number of overlapped DEGs divided by the number of DEGs from the comparison with a smaller number DEGs. (b). Fold correlation for SMART_CC and SMART_Nxt common DEGs

**Figure S6. Number of differentially expressed genes decreased with reduced input**

(a) Number of detected DEGs between α-CD3 and α-CD3 + B7-1-Fc at different cell inputs in each protocol using the common DEGs detected by all three protocols at 100K cells as reference (FDR<0.05). The absolute number of detected genes were shown

above each bar in the plot. (b) Sensitivity for detecting common DEGs at low input samples.

## Supplemental Table 1-6

Supplemental Table 1: qRT_PCR validation for AmpliSeq specific DEGs
Supplemental Table 2: qRT_PCR validation for SMART_CC and AmpliSeq DEGs
Supplemental Table 3: qRT_PCR validation for SMART_CC specific DEGs
Supplemental Table 4: qRT_PCR validation for SMART_Nxt and AmpliSeq DEGs
Supplemental Table 5: qRT_PCR validation for SMART_Nxt and SMART_CC DEGs
Supplemental Table 6: qRT_PCR validation for SMART_Nxt specific DEGs

## Supplemental Table 1

| | LogFC_2 | | | | |
|---|---|---|---|---|---|
| GeneID | Ampliseq | Clontech | Nextera | Type | Fluidgm Fold change |
| ARHGEF26 | -4.451 | -0.237 | -0.174 | ampliseq_only | 4.729711104 |
| ARHGEF35 | 2.778 | -0.116 | -0.214 | ampliseq_only | 0.070400418 |
| CACNA2D2 | -2.01 | -0.319 | -0.609 | ampliseq_only | 0.216487808 |
| CACNA2D4 | -2.725 | -0.255 | -0.345 | ampliseq_only | 0.403306269 |
| CCL3L3 | 8.098 | 0 | 0 | ampliseq_only | 3547.935556 |
| CRLF2 | 2.799 | 0 | 0.028 | ampliseq_only | 1575.137645 |
| ENTPD1 | 2.176 | 0.231 | 0.075 | ampliseq_only | 1734.013322 |
| FARP1 | 2.616 | -0.102 | 0.28 | ampliseq_only | 0.126832463 |
| FCRL3 | -2.07 | -0.509 | -0.652 | ampliseq_only | 2.53225905 |
| GRK4 | 5.384 | -0.508 | -0.547 | ampliseq_only | 0.975671779 |
| IKBKG | 4.02 | 0.196 | 0.12 | ampliseq_only | 0.555741 |
| IL3RA | 2.027 | 0.149 | 0.657 | ampliseq_only | 157.587791 |
| KIR3DL2 | 2.769 | 0.648 | 0.566 | ampliseq_only | 35.34443274 |
| LGALS9C | -3.26 | 0.024 | -0.884 | ampliseq_only | 0.304111852 |
| MAK | 2.316 | -0.013 | 0.464 | ampliseq_only | 195.6126115 |
| MDP1 | -4.833 | -0.123 | -0.126 | ampliseq_only | 0.714284957 |
| PPP1R2P3 | -4.368 | 0.005 | 0.071 | ampliseq_only | 0.000318083 |
| PTPN14 | 4.884 | 0.325 | -0.197 | ampliseq_only | 2416.565431 |
| RGS5 | -2.841 | -0.234 | 0.002 | ampliseq_only | 12.36557016 |
| SPDYA | -4.62 | -0.333 | -0.337 | ampliseq_only | 33.51900148 |
| TERT | -4.619 | 0.366 | 0.035 | ampliseq_only | 8.2107227 |
| USP17L5 | -2.736 | 0 | 0 | ampliseq_only | 0.018428531 |

~59% match

## Supplemental Table 2

| | LogFC_2 | | | | |
|---|---|---|---|---|---|
| GeneID | Ampliseq | Clontech | Nextera | Type | Fluidgm Fold change |
| ABCA6 | -2.057 | -2.543 | 0.348 | clontech_ampliseq | 157.5708718 |
| CCR5 | 3.558 | 2.346 | 0.668 | clontech_ampliseq | 0.223984855 |
| HIPK4 | -2.229 | -3.013 | -0.365 | clontech_ampliseq | 0.312898872 |
| LIPH | 6.74 | 2.686 | 0.673 | clontech_ampliseq | 2471.645148 |
| SRGAP1 | -3.562 | -2.298 | -0.459 | clontech_ampliseq | 0.126832463 |
| TEX29 | -2.321 | -2.183 | -0.458 | clontech_ampliseq | 0.910199088 |

~66% match

## Supplemental Table 3

| | LogFC_2 | | | | |
|---|---|---|---|---|---|
| GeneID | Ampliseq | Clontech | Nextera | Type | Fluidgm Fold change |
| ARSD | -0.578 | -2.412 | -0.813 | clontech_only | 2.041292716 |
| CKAP2L | 0.965 | -2.686 | -0.178 | clontech_only | 7.534788593 |
| ENPP3 | -0.663 | 2.513 | 0.899 | clontech_only | 73.92026442 |
| FAM64A | -0.6 | -5.197 | -0.784 | clontech_only | 0.126832463 |
| FTH1P3 | 0.327 | -2.068 | -0.084 | clontech_only | 0.083361652 |
| GALNT8 | -0.508 | 4.87 | 0.035 | clontech_only | 0.927793753 |
| IQCA1 | 0.049 | -2.173 | -0.951 | clontech_only | 6.110590983 |
| JAKMIP2 | -0.743 | -2.364 | -0.538 | clontech_only | 26.64622903 |
| NOSTRIN | -0.857 | -3.014 | 0.033 | clontech_only | 0.775658733 |
| PHKA1 | -0.723 | -3.464 | -0.949 | clontech_only | 0.05186038 |
| POTEE | 0 | 3.092 | 0.033 | clontech_only | 1.132802248 |
| SEMA5A | -0.448 | -2.656 | -0.944 | clontech_only | 8.172515188 |
| SLC45A3 | -0.191 | -2.061 | -0.908 | clontech_only | 15.82384763 |
| TSPAN1 | -0.472 | -5.345 | -0.658 | clontech_only | 0.126832463 |

~50% match

## Supplemental Table 4

| | LogFC_2 | | | | |
|---|---|---|---|---|---|
| GeneID | Ampliseq | Clontech | Nextera | Type | Fluidgm Fold change |
| ACOT12 | -2.671 | -0.376 | -3.206 | nextera_ampliseq | 4.126932578 |
| CSF2RA | 3.9 | 0.869 | 4.875 | nextera_ampliseq | 20167.20544 |
| GPR156 | -2.18 | -0.659 | -2.394 | nextera_ampliseq | 255.6361657 |
| MPL | 3.079 | 0.337 | 2.722 | nextera_ampliseq | 87.21847306 |
| PLOD2 | 3.34 | 0.995 | 2.264 | nextera_ampliseq | 707.0014277 |
| PROC | 4.669 | 0.924 | 3.542 | nextera_ampliseq | 0.126832463 |
| TUBB2B | -6.632 | -0.592 | -2.921 | nextera_ampliseq | 0.015163991 |

~57% match

## Supplemental Table 5

| | LogFC_2 | | | | |
|---|---|---|---|---|---|
| **GeneID** | **Ampliseq** | **Clontech** | **Nextera** | **Type** | **Fluidgm Fold change** |
| CCR8 | -0.894 | -3.764 | -2.444 | nextera_clontech | 0.328639365 |
| CLDN5 | 0 | 6.726 | 4.329 | nextera_clontech | 59.04443075 |
| DDAH1 | 0.05 | -2.64 | -2.729 | nextera_clontech | 350.542172 |
| G0S2 | 0.21 | 3.315 | 4.875 | nextera_clontech | 223.0889748 |
| GNG4 | 1.691 | 2.48 | 2.61 | nextera_clontech | 11.82291923 |
| IGF2BP3 | 1.424 | 2.136 | 1.668 | nextera_clontech | 168.6752421 |
| LRRC32 | 0 | 5.728 | 8.27 | nextera_clontech | 627.3610374 |
| MPP3 | -0.869 | -3.241 | -2.333 | nextera_clontech | 0.700955433 |
| MYLK | 0.047 | 3.345 | 3.141 | nextera_clontech | 12595.5048 |
| NPC1L1 | -1.639 | -2.894 | -2.57 | nextera_clontech | 1975.647765 |
| PDGFRA | 0 | 5.74 | 3.978 | nextera_clontech | 1223.588505 |
| SORBS1 | 0 | -2.471 | -2.833 | nextera_clontech | 0.158461889 |
| TBC1D8B | -1.459 | -2.841 | -1.292 | nextera_clontech | 24.85857359 |
| TMSB4Y | -0.561 | -4.458 | -7.717 | nextera_clontech | 0.029909375 |
| TNFSF14 | 0 | 5.407 | 5.274 | nextera_clontech | 11204.03815 |
| TNS1 | 1.017 | 2.963 | 5.241 | nextera_clontech | 0.0463417 |
| ZBTB32 | 0.042 | 2.522 | 2.653 | nextera_clontech | 1.535469451 |

~76% match

## Supplemental Table 6

| | LogFC_2 | | | | |
|---|---|---|---|---|---|
| GeneID | Ampliseq | Clontech | Nextera | Type | Fluidgm Fold change |
| CPLX3 | -0.447 | 0.022 | -4.375 | nextera_only | 0.531113727 |
| NDUFA4L2 | 0.723 | 0.554 | 4.877 | nextera_only | 8.528112234 |
| PHEX | -0.488 | 0.911 | 2.932 | nextera_only | 126.586083 |
| RDH16 | 0.477 | 0.307 | -2.904 | nextera_only | 176.3005933 |
| RPS26P11 | -0.482 | 0.355 | -5.027 | nextera_only | 0.095360996 |
| RUSC2 | 0.675 | -0.27 | -2.48 | nextera_only | 3.515482612 |
| SIGLEC9 | -0.196 | 0.478 | 2.402 | nextera_only | 2284.503158 |
| SNORA58 | 0.23 | 0.715 | 3.324 | nextera_only | 0.992248372 |
| YY2 | 0.103 | 0.985 | 2.22 | nextera_only | 1.936806054 |
| ZBTB8A | -0.356 | -0.875 | 2.357 | nextera_only | 4.974363037 |
| ZNF221 | 0.577 | 0.705 | 2.234 | nextera_only | 1.208235059 |

~72% match
